# CVD Mortality Disparities with Risk Factor Associations Across U.S. Counties

**DOI:** 10.3390/healthcare13222937

**Published:** 2025-11-17

**Authors:** David H. An

**Affiliations:** Harvard University, Massachusetts Hall, Cambridge, MA 02138, USA; davidan@college.harvard.edu; Tel.: +1-614-705-9654

**Keywords:** cardiovascular disease, risk factors, geospatial distribution, correlation, statistical modeling

## Abstract

Introduction: Cardiovascular disease (CVD) remains a primary cause of mortality worldwide, with persistent geographic disparities driven by a complex interplay of risk factors. Continual updates of localized variations in CVD mortality are essential to develop targeted interventions for optimizing disease and healthcare management. Methods: This study investigated associations between CVD mortality and a comprehensive set of biological, environmental, behavioral, and socioeconomic factors across all U.S. counties, employing correlation, geospatial visualization, stepwise multiple regression, and machine learning models to evaluate the importance of risk associations. Results: Significant disparities in CVD mortality trend were observed across race, age, sex, and region, with elevated rates among older adults, men, and Blacks, particularly in southeastern states exhibiting severe social vulnerability. Correlation analysis identified disease management (e.g., COPD, hypertension, medication non-adherence), environmental factors (PM2.5), lifestyle behaviors (e.g., smoking, sleep duration), and socioeconomic status (e.g., poverty, single-parent households, education) as important contributors to CVD mortality. Conversely, higher household income, physical activity, and cardiac rehabilitation participation were strong protectors. Multiple regression explained 66.9% variance in CVD mortality, recognizing PM2.5, smoking, and medication non-adherence as top associated factors. Random Forest models underscored COPD’s predictive dominance, followed by medication non-adherence, smoking, and sleep duration. Conclusions: The findings highlight the geospatial connection of risk factors to CVD mortality disparities across U.S. counties. They emphasize the critical importance of data-driven strategies targeting air quality, tobacco control, social inequities, and chronic disease management to mitigate CVD burden and promote health equity.

## 1. Introduction

Cardiovascular disease (CVD) has been acknowledged as posing one of the foremost challenges to global public health in the 21st century [1]. It claims millions of lives annually, with nearly 80% of deaths occurring in lower-income countries, reflecting disparities in societal and economic development. Even in the U.S., CVD remains a primary healthcare concern despite extensive prevention and treatment advancements [1]. Projections indicate a rise in prevalence and associated costs through 2050 due to population aging and persistent risk factors, especially within racially diverse communities [2]. While improvements in medical technology and healthcare have reduced age-relevant mortality, regional and social disparities in CVD outcomes persist. Understanding the regional distribution and impact of diverse risk factors is crucial for designing targeted public health interventions.

Multiple risk factors, including hypertension, diabetes, obesity, smoking, physical inactivity, and insufficient sleep, have been consistently recognized as biological and behavioral determinants of adverse cardiovascular outcomes [1,2,3,4,5]. Beyond these traditional factors, emerging evidence highlights environmental and social determinants, such as air pollution exposure (PM2.5) [6], heavy metal contamination [7], transportation noise [8], limited green spaces [9], and socioeconomic disadvantage [10,11,12], in exacerbating CVD risk across regions and populations. Particularly, household socioeconomic status, encompassing education, income, and Internet access, has been linked to cardiovascular health, indicating systemic inequities in medical access and information [10,13,14,15].

The interplay among these determinants is complex and often synergistic. More than half of CVD cases may result from the combined impact of five major modifiable factors [5]. This intricate interplay often fosters bidirectional relationships between CVD and other conditions, such as cancer, mental disorders, and chronic obstructive pulmonary disease (COPD) [12,16]. Moreover, the relationships between the interrelated risk factors and CVD outcomes vary across populations and geographic areas [10,11,17]. This underscores the necessity for geographically fine-grained insights into their associations to inform targeted public health interventions.

Numerous studies have explored CVD risk and factor associations across various geographic scales. Specifically, state-level analyses in Georgia identified household income and PM2.5 as the leading contributors [18], while national studies emphasized demographic composition, education, income inequality, and social vulnerability [19]. Research from South Korea linked high CVD mortality to poor air quality and insufficient green infrastructure [9]. Cross-national studies suggest that CVD mortality can be possibly affected by surrounding countries, with income and other socioeconomic variables being key influencers [14]. Despite these efforts, most analyses have relied on geographical averages or limited variable sets, often evaluating individual factors in isolation. There remains a critical need for geographically resolved analyses that integrate all the relevant factors simultaneously into consideration [2].

The absence of such integration has precluded measuring factors’ joint effects or spatial interactions, thereby constraining our understanding of CVD disparities at a fine-grained geographic level. To address this gap, the present study conducted a county-level analysis across all U.S. regions to capture the complex interdependencies between CVD mortality and a comprehensive set of risk factors. The study’s novelty resides in its high-resolution integration of multi-domain variables, covering traditional, environmental, behavioral, and socioeconomic perspectives, within a unified, data-driven framework. This framework significantly advances prior work by simultaneously evaluating the comprehensive set of factors through a combination of conventional statistical techniques and machine learning (ML) models and visualizing their geospatial relationships.

Accordingly, this study aimed to (1) investigate macro-scale geographic associations between CVD mortality and a complex mixture of human, social, and environmental factors; (2) visualize the spatial distribution of CVD mortality and risk factors across U.S. counties; (3) employ advanced statistical and ML methods to analyze complex interdependencies; (4) inform data-driven and targeted intervention strategies to reduce CVD mortality and promote health equity. Ultimately, the findings will contribute to a more comprehensive understanding of the multifactorial and geographically heterogeneous nature of CVD mortality in the United States.

## 2. Methodology

### 2.1. Data Collection

The data used for analyses were collected from publicly accessible databases of the Centers for Disease Control and Prevention (CDC) on 21 November 2024. These were organized in national-by-county geographic type encompassing CVD mortality rates and other different variables. The CVD mortality rates, expressed as the number of total vascular disease deaths per 100,000 population, were sourced from the CDC Heart Disease & Stroke Interactive Atlas (http://nccd.cdc.gov/DHDSPAtlas/Reports.aspx (accessed on 21 November 2024)). This dataset spanning the period 2006–2021 was disaggregated by age, gender, and ethnicity, allowing for longitudinal analysis of mortality trends before and during the COVID-19 pandemic across the U.S. Other datasets were gathered from the National Environmental Public Health Tracking Network (https://ephtracking.cdc.gov/download (accessed on 21 November 2024)) or the CDC National Center for Health Statistics (NCHS) (https://data.cdc.gov/browse (accessed on 21 November 2024)), wherever they were available.

Specifically, drug poisoning mortality rates were downloaded from NCHS; Interactive Atlas was used to extract relevant data pertaining to lifestyle prevalence of coronary heart disease, high blood pressure, stroke, high cholesterol, diabetes, obesity, physical inactivity, alcohol use, insufficient sleep, and smoking status, social and economic status of broadband Internet, computer ownership, education, food stamp, median home value, median household income, income inequality, poverty, housing cost burden, and unemployment rate, physical environment of air quality, park access, and urbanization, and healthcare delivery status of insurance coverage, care costs, blood pressure medication, diuretic non-adherence, renin-angiotensin system antagonism non-adherence, cholesterol-lowering medication, cholesterol screening, cardiac rehabilitation, hospitals, pharmacies, physicians, and specialists; Tracking Network was utilized for the collection of factors and indicators including prevalence of asthma, cancer, and COPD, demographic and socioeconomic indices in the composition of community capital resilience, economic resilience, environmental resilience, infrastructural resilience, institutional resilience, social resilience, and social vulnerability, individual components like household composition, transportation, single family, etc., and built environment like age of housing, land cover and use, traffic safety, sunlight, exposure to hazards, etc.

To ensure temporal consistency across all domains in correlation and statistical modeling analyses, both CVD mortality and exposure variables were averaged over a three-year period (2019–2021). This timeframe was chosen to minimize the influence of short-term fluctuations and anomalous events, such as the immediate COVID-19 shock in 2020, while still capturing contemporary conditions reflective of current CVD risk patterns.

### 2.2. Data Preparation

The gathered datasets contained either FIPS (federal information processing standards code uniquely identifying counties in the U.S.) or county-state geographic information in their dataframe. For datasets missing FIPS, a geographic identifier was added by cross-checking county-state information with a standardized code reference table via a Python-based approach. The individual datasets were then merged into a single unified data-frame using the ‘FIPS’ code as the primary index key through the data manipulation library of Pandas in Python. This allowed for the integration of data from various sources into a coherent structure for subsequent analyses. For regression and ML modeling described below in the Data Analyses subsection, entries with missing values (NA) were removed via listwise deletion to maintain analytical integrity and statistical reliability. This approach was chosen instead of imputation because the proportion of missing data was not clustered within particular variables, minimizing the risk of bias. It also prevented the introduction of artificial variance or assumptions about the missing patterns that could distort real associations. Future research with denser or individual-level data could benefit from imputation strategies; however, for the present county-level dataset, deletion provided the most transparent and replicable method. Scatter plot exploratory data analyses (EDAs) were initially performed to visualize the distribution of the variables of interest. This visual inspection helped identify the need for data transformation. Box–Cox transformation was considered to stabilize variance if the scatter plot indicated a non-normal distribution or heteroscedasticity, otherwise, Johnson transformation was explored as an alternative. At this stage, twenty of the 111 total variables (18%) were transformed to ensure comparable scale and variance prior to data analysis.

### 2.3. Data Analyses

The study was structured with counties as the units of analysis. One-way ANOVA was used to determine differences in CVD mortality across race, age, gender, and year. This initial step allowed for the identification of potential trends and disparities in CVD mortality over time and across demographic groups. To evaluate risk factors influencing CVD mortality, a stepwise analytical framework was employed, as detailed below.

Correlation analysis was first utilized to examine relationships between CVD mortality and exposure variables. Pearson’s correlation coefficient was calculated to measure the strength and direction of linear associations from the variables. The factors exhibiting low to strong correlation with CVD mortality were selected for later statistical modeling and visualization. Geographic information systems (GISs) were used to map the spatial distribution of CVD mortality and selected risk factors. This enabled the visualization of regional disparities in CVD mortality and risk factor associations across U.S. counties.

Multiple regression analysis employing stepwise selection of least squares was utilized to estimate the independent and combined effects of the chosen factors on CVD mortality. Variables with high collinearity or non-significant effects (*p*-value > 0.05) were excluded to enhance regression efficacy. This framework accounted for potential confounders and interactions among variables to ensure robust inference. To assess regression robustness, 10-fold cross-validation was performed on the final model. The model adequacy was further visualized through the spatial mapping of residuals at both the county and state levels, allowing for a geographic diagnosis of under- or over-estimation.

To complement the regression analysis, two popular ML models, Random Forest (RF) and Support Vector Machine (SVM), were implemented to better capture potential nonlinear relationships. This dual-model strategy enabled a comparative and complementary assessment of predictive performance: RF provided feature importance metrics, while SVM served as a nonlinear benchmark to validate observed relationships. Hyperparameters for the RF configuration included n_estimators = 500, max_depth = none, min_samples_split = 2, min_samples_leaf = 1, max_features = ‘sqrt’, bootstrap = True, random_state = 42, and n_jobs = −1. For SVM, the parameters were kernel = ‘rbf’, C = 10, epsilon = 0.1, and gamma = ‘scale’. The dataset was randomly partitioned into 80% for training and 20% for subsequent evaluation. Fivefold cross-validation within the training set was used to provide additional error control for ML performance. Model performance was assessed with Mean Squared Error (MSE) and R-squared (R^2^) for effective prediction.

All analyses were performed in Python (v 3.11) using open-source libraries: pandas (2.2.2) and numpy (1.26) for data processing, scikit-learn (1.4.2) for ML algorithms and metrics, and matplotlib (3.8) and seaborn (0.13) for visualization. Together, the comparative-complementary design of statistical inference and predictive modeling approaches would enable cross-validation of the results. The convergence of key variables across these frameworks will enhance analytical confidence toward a unified understanding of the risk associations of CVD mortality. The inclusion of spatial residual mapping will assist analysis transparency, offering a comprehensive understanding of the geographic and multifactorial determinants of CVD mortality across the U.S.

## 3. Results

### 3.1. Demographic Disparities

**Figure 1:**
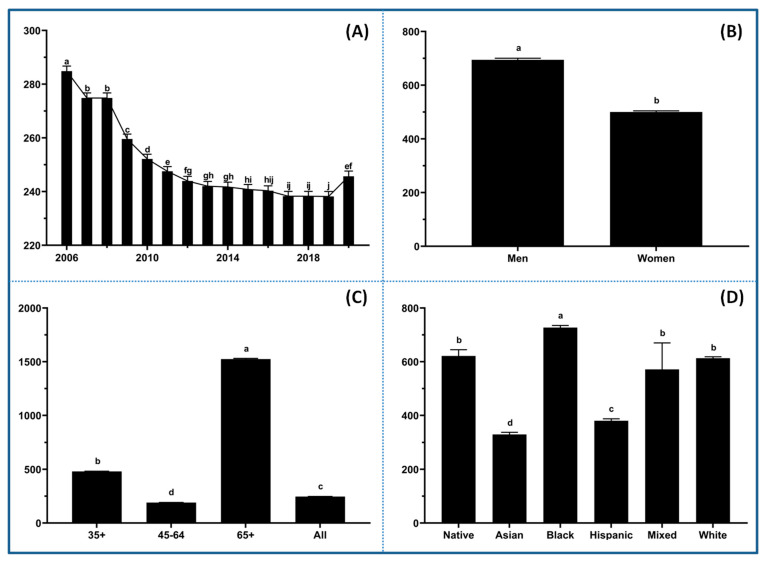
CVD mortality trends across various demographic groups. (**A**) The fluctuations in CVD mortality rates over a 12-year period from 2006 to 2018. (**B**) Comparison of CVD mortality rates between men and women. (**C**) Comparison of CVD mortality rates across different age groups. (**D**) Comparison of CVD mortality rates among various racial and ethnic groups. The letters (a, b, c, etc.) on the graphs indicate statistical significance between groups.

The data were initially explored to visualize trends and disparities in CVD mortality across racial, age, and gender groups before diving deeper into associated risk factor profiles. There was a notable decline in mortality rates from 2006 to 2019 (Figure 1A), indicating the effectiveness of public health interventions and advancements in medical treatment. However, the trend bounced back in 2020 and reached similar levels as in 2011 and 2012, implying that COVID-19-related factors had a significant impact on CVD mortality. Significant disparities in CVD mortality were observed between men and women (Figure 1B). Men have consistently exhibited higher rates of mortality compared to women across all age groups. There is a strong association between age and CVD mortality, with the highest death rates occurring in the 65+ age group (Figure 1C), emphasizing the critical need for early prevention and risk management strategies. The data also revealed stark disparities in CVD mortality across racial and ethnic groups (Figure 1D), with Black populations experiencing the highest rates, followed by Native American, White, Mixed, Hispanic, and Asian. It is not surprising that mixed race groups showed the highest variation in CVD mortality compared to others. The pronounced disparities across racial and ethnic groups might reflect long-standing inequities in healthcare access, socioeconomic conditions (e.g., poverty, food insecurity, inadequate housing, unemployment, etc.), and exposure to other risk factors such as hypertension, diabetes, smoking, mental health, and environmental toxins [20,21]. To address disparities across different demographic groups, it is essential to conduct further research integrating comprehensive behavioral, socioeconomic, and environmental data into cardiovascular health strategies to develop targeted interventions [20].

### 3.2. Key Correlation Factors

The correlation analyses between CVD mortality and various factors categorized their associations into different significances. Conventionally, correlation was considered as strong (absolute R ≥ 0.7), moderate (0.5 ≤ absolute R < 0.7), low (0.3 ≤ absolute R < 0.5), and negligible (absolute R < 0.3) [22]. The strong to low correlations identified in this study are summarized in Table 1, while the full matrix is available in the Supplementary Materials (Table S1). A large portion of factors had *p*-values ≤ 0.0001, indicating their significant relationships with CVD mortality.

**Figure 2:**
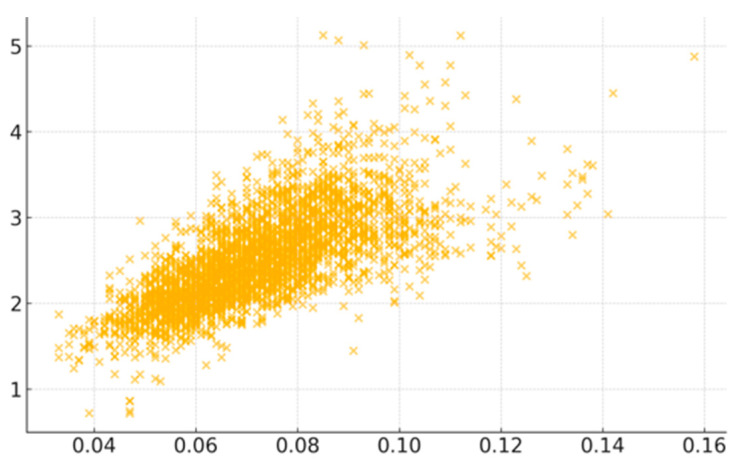
Scatter plot displaying the correlation between COPD and CVD mortality across the U.S. counties.

Among the risk factors, COPD exhibited the strongest positive correlation with CVD mortality. The scatter plot visually confirms COPD’s significance (Figure 2), but the moderate spread of data points also reflects the multifactorial nature of CVD risk. Smoking was the second most highly associated factor, showing moderate correlation, which underlines the critical importance of tobacco control in public health management. Likewise, high blood pressure as a direct contributor to CVD helps explain its correlation, as well as stroke. Less sleep emerged as another significant risk factor, possibly linked to chronic stress and hypertension [23]. Indicators of socioeconomic disadvantages (e.g., poverty rate and reliance on food assistance programs) were moderately correlated with increased CVD mortality. Together with unhealthy lifestyles and stress, they indicate how limited healthcare access and chronic stress exacerbate CVD risk.

Social vulnerability indicators like vulnerability rank, single-parent households, and vulnerability indices displayed low but significant correlations, linking the impact of social determinants on health outcomes. Health-related behaviors and factors like coronary heart disease, diabetes, physical inactivity, and non-adherence to medications were weakly associated, reflecting their contribution to CVD mortality. The low R values for surrounding environment like household Internet access and PM2.5 indicate that living conditions have a considerable impact on cardiovascular health.

Interestingly, obesity, though a well-established health risk factor, demonstrated only a negligible correlation (R = 0.09; *p*-value < 0.0001), suggesting more nuanced relationships. Conversely, factors like median household income, social resilience, alcohol use, and institutional resilience with negative correlations indicate low to moderate protective effects. While it has been inconclusive and conflicting as to whether alcohol consumption offers cardioprotection in previous research [24], this study favors its protective effects. The negative correlation of households with smartphones implies that there is a weak to negligible protective relationship with CVD mortality. Given the high penetration of smart phones into groups with low socioeconomic status, health-related mobile applications might provide an opportunity to overcome traditional barriers to cardiac rehabilitation access [25].

In terms of accessibility, factors related to urbanization and healthcare infrastructure, such as availability of cardiac rehabilitation hospitals and walkability, were negligibly but inversely linked to reduced CVD mortality, reflecting marginal benefits of improved healthcare access and active transportation. Surprisingly, the geospatial correlation barely favored a close relationship between CVD and cancer, despite being two of the leading causes of death worldwide with known common mechanisms and risk factors that predispose individuals to both conditions [12]. This negligible correlation, though statistically significant, suggests that their interconnection is not reflected at a geographic level, a phenomenon also observed in the case of obesity.

Finally, there were a few factors like atrazine in water and certain healthcare access measures (e.g., cardiovascular physician) that showed no significant relationships (*p*-value > 0.05). This is in conflict with a previous meta-analysis that indicated the positive association of CVD with chronic exposure to drinking water arsenic at concentrations below the WHO provisional guideline value [26]. One explanation could be the limited data points for this factor in the study. Collectively, these correlation patterns provide an empirical foundation for subsequent multivariable regression and ML modeling.

### 3.3. Geospatial Visualization

Three correlation factors at moderate (social vulnerability index—SVI), low (air quality PM2.5), and negligible (sunlight UV exposure) levels were chosen to showcase the geospatial disparities and potential environmental and social contributors to cardiovascular health outcomes across the U.S.

**Figure 3:**
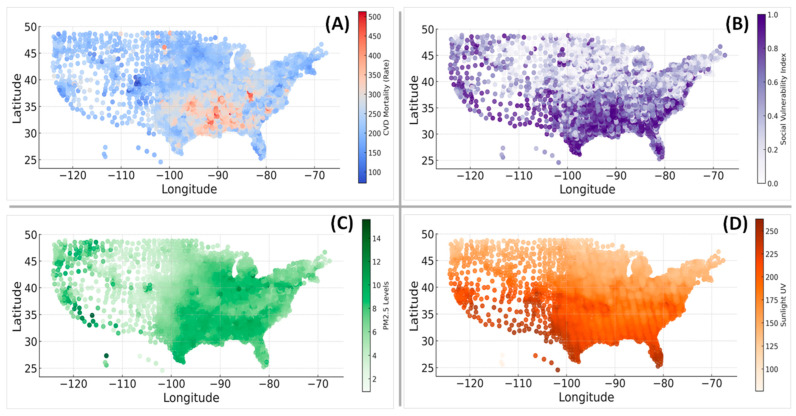
Spatial patterns of CVD mortality and risk factors across the U.S. counties. (**A**) Spatial distribution of CVD mortality rates (per 100,000) with higher depicted in red and lower in blue. (**B**) Social vulnerability index (SVI) scores ranging from low (white) to high (dark purple). (**C**) Average annual air quality PM2.5 concentrations represented by shades of green, highlighting regions with different levels of air pollution. (**D**) Sunlight UV index distribution with darker orange indicating higher exposure levels.

The geographic distribution revealed regional disparities with high CVD mortality rates prominently visible in the southeastern U.S., particularly around the Mississippi River Basin, including states like Mississippi, Alabama, Louisiana, and parts of Arkansas (Figure 3A). In contrast, areas in the western and northeastern U.S. displayed significantly lower mortality. These disparities underscore the potential role of public health interventions targeting high-risk regions to reduce CVD burden.

The SVI, as a composite measure of community resilience to external stressors such as natural disasters, economic shocks, or public health crises, demonstrated strong spatial alignment with CVD mortality (Figure 3B). High SVI scores, reflecting populations facing poverty, limited access to education and healthcare, and transportation challenges, were predominantly concentrated in the southern and southeastern regions. The geographic overlap between elevated SVI and high CVD mortality underscores how social vulnerabilities can exacerbate cardiovascular risk and worsen outcomes. Conversely, lower SVI scores in the Midwest and western states suggest stronger social and economic resilience, likely correlating with favorable cardiovascular outcomes.

Higher concentrations of PM2.5 were observed in regions with significant industrial activity, urbanization, or reliance on fossil fuel combustion, such as parts of California, the Midwest (including the Ohio River Valley), and the Northeast (Figure 3C). Long-term exposure to PM2.5 has been linked to cardiovascular conditions, including atherosclerosis, hypertension, and myocardial infarction due to mechanisms such as systemic inflammation and oxidative stress [27]. While there was no notable visual overlap between poor air quality and high CVD mortality, many regions with high PM2.5 levels coincided with elevated death rates, indicating that chronic environmental exposures might amplify existing health disparities.

The distribution of annual sunlight exposure across the U.S. demonstrated a clear latitudinal gradient, with higher levels in southern states (Figure 3D). Sunlight has complex effects on health, with moderate exposure promoting vitamin D synthesis but excessive exposure increasing oxidative stress and inflammation, potentially influencing cardiovascular health. The southern regions, experiencing both higher sunlight intensity and elevated CVD mortality, may thus represent areas where climatic and behavioral factors interact to influence outcomes.

Taken together, the spatial patterns illustrate that CVD mortality is highly heterogeneous across the U.S., shaped by a convergence of social, environmental, and geographic factors. The high CVD mortality occurring in regions with social and environmental vulnerability highlights the critical need for location-based public health strategies that address multiple determinants simultaneously.

### 3.4. Multiple Regression Modeling

Variables with absolute R values ≥ 0.3 in the correlation were subjected to a stepwise multiple regression analysis. Factors with non-significant *p*-values (>0.05) or multicollinearity concerns in the regression were sequentially excluded to optimize model performance. During resolution of the multicollinearity, Principal Component Analysis (PCA) was applied to interrelated variables. The first principal component (PC1) from the PCA of COPD, coronary heart disease, smoking, high blood pressure, and stroke initially showed a variance inflation factor (VIF) value greater than 5, suggesting strong correlation and synergistic effects among these factors. Through stepwise removal and comparison of R^2^ and VIF values, smoking status was retained as it provided the optimal model balance (higher R^2^ and lower VIF). After optimization, all variables participating in the final regression fell below the VIF threshold of 5, confirming that multicollinearity was adequately resolved.

The optimized regression model achieved R^2^ of 66.93%, closely matching the adjusted (66.72%) and predicted (66.34%) values (Table 2). This suggests that the model has moderate predictive ability without over fitting. The regression coefficients and t-values further pointed to the strength and significance of the associations. The analysis highlighted the interplay of socioeconomic, behavioral, and environmental factors influencing CVD mortality. Particularly, PM2.5 emerged as the strongest factor, being followed by smoking status, blood pressure medication (BPM) non-adherence, cardiac rehabilitation eligibility, and blood pressure medication. These results highlight modifiable behavioral and clinical targets for CVD prevention.

Socioeconomic factors, such as single-parent households, food stamp usage, no college degree, and disability, were positively associated with CVD mortality. Other lifestyle and health-related factors, such as diabetes, sleep duration, and post-acute care cost, were also significant. Protective socioeconomic factors such as household income, park access, and mobile-home housing offer opportunities for structural improvements in cardiovascular health outcomes. Factors like no high school diploma and alcohol use were inversely associated with CVD mortality rates, potentially reflecting confounding lifestyle variables. Interestingly, factors of no college degree and no high school diploma contributed to the regression model differently with one positive and the other negative. This divergence might be attributed to the broader trend and association between higher education and healthier lifestyles. Individuals with higher educational backgrounds are less prone to risk factors like smoking, high salt intake, air pollution exposure, and depression. Conversely, they are more likely to engage in physical activity and benefit from increased household income, highlighting the importance of mitigating educational inequality in efforts to address CVD mortality disparities [28].

**Figure 4:**
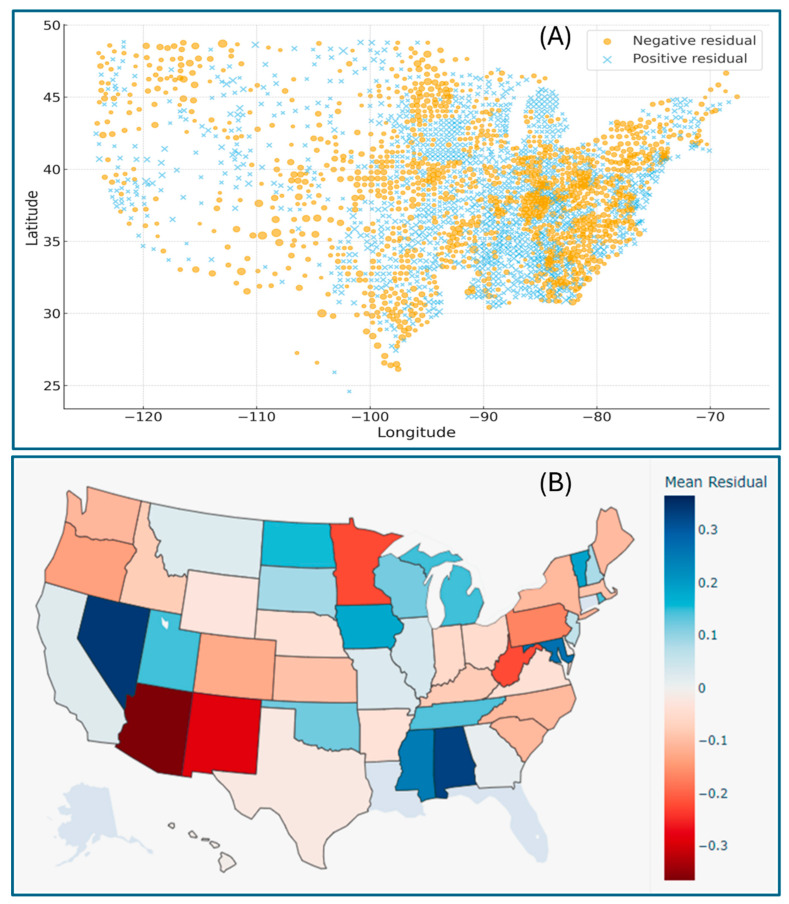
Geographic distribution of regression residuals across the United States. (**A**) The spatial patterns present the county-scale residual distribution using individual data points located at each county’s centroid. Marker size is proportional to the absolute residual value. Marker shape differentiates residual direction, with ‘X’ corresponding to positive residuals or underestimation and ‘O’ to negative residuals or overestimation. (**B**) The spatial patterns illustrate the state-level mean residuals from the multiple regression model, averaged across all counties within each U.S. state. The diverging color scale indicates underestimation (positive residuals in red tones) or overestimation (negative residuals in blue tones) of the observed CVD mortality by the regression model, and the color intensity reflects the magnitude of bias.

Geographic distribution maps of the regression residuals further visualized the spatial heterogeneity of model performance across the U.S., clearly indicating where the regression fit between the CVD mortality and exposure variables deviated. The county-level patterns pinpoint the model performance, indicating that socio-environmental or demographic factors unexplored in this study may contribute to the observed systematic deviation (Figure 4A). The state-level distribution of larger positive residual clusters in certain areas (e.g., Nevada, Alabama, Mississippi, and Maryland) suggests the localized underestimation of CVD mortality, whereas regions with significant negative residuals (e.g., Arizona, New Mexico, Minnesota, and West Virginia) imply potential overestimation (Figure 4B). These spatial trends reveal the importance of incorporating regional and contextual variables in future modeling frameworks.

### 3.5. Machine Learning Prediction

The RF and SVM models were tested to evaluate the predictive power of various factors on CVD mortality. Both models demonstrated strong predictive performance in estimating county-level CVD mortality, with results closely aligned with the correlation and regression analyses. The RF model achieved a higher overall predictive accuracy (MSE = 0.0984, R^2^ = 0.696), outperforming the SVM model (MSE = 0.1119, R^2^ = 0.654). The performance gap reflects RF’s superior ability to capture complex feature interactions and handle nonlinearities in this study. The comparative evaluation of RF and SVM performance underscores their mutual validity and complementarity in modeling CVD mortality across U.S. counties. RF effectively captures nonlinear, multicollinear, and interaction effects among predictors. SVM, though slightly less accurate, confirms that the relationships identified by RF are structurally consistent. The close alignment between SVM and RF outcomes strengthens the confidence in identified determinants.

**Figure 5:**
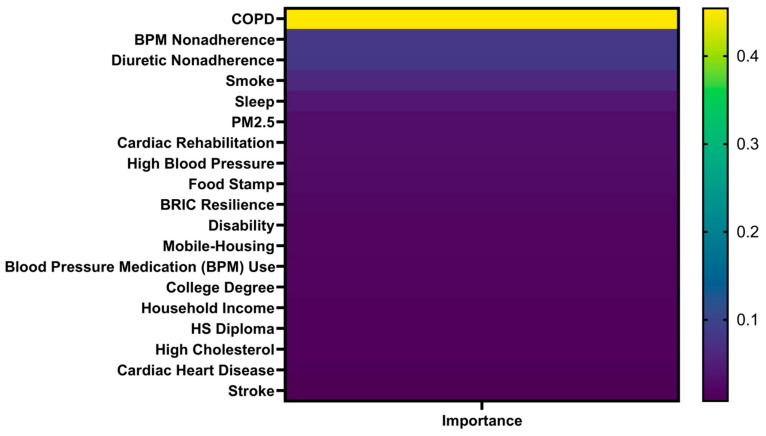
Dominance of risk factors in predicting CVD mortality with random forest models across the U.S. counties.

Feature importance estimates from RF weighted the relative contributions of individual factors to the prediction of CVD mortality. COPD emerged as the most critical factor, contributing nearly half (45.42%) of the model’s predictive power (Figure 5). Following COPD, the most influential factors included non-adherence to blood pressure medications, diuretic non-adherence, smoking, sleep duration, and PM2.5 exposure. In addition, cardiac rehabilitation and high blood pressure were notable features in the model. This highlights the critical role of medication adherence in maintaining cardiovascular health, especially for patients on first-line treatment of hypertension and heart failure, as non-adherence can lead to fluid retention, increased cardiac workload, and higher risks of stroke [29]. In terms of SVM’s prediction, it produced a comparable rank ordering of key variables, whereas its nonlinear kernel-based architecture could not provide interpretable feature weights, limiting transparency in understanding factor contributions. Nevertheless, both models demonstrated high consistency in identifying respiratory and behavioral factors as dominant contributors, reinforcing the reliability of the analytical framework.

## 4. Discussion

This study presented a comprehensive analysis of CVD mortality and its associations with a wide spectrum of risk factors across the U.S. counties. A rigorous stepwise analytical framework, integrating correlation, regression and ML, was employed to identify and prioritize risks for targeted interventions. The multi-stage approach ensures that variables with subtler effects are not overshadowed by stronger factors in a multivariate context, thereby enhancing the comprehensiveness and reliability of the analytical results. The findings are broadly consistent with large national and international investigations, including the Global Burden of Disease (GBD), Institute for Health Metrics and Evaluation (IHME), and American Heart Association (AHA) studies, which collectively emphasize the roles of air pollution, smoking, hypertension, and socioeconomic deprivation as principal factors of CVD mortality [1,2]. Whereas GBD studies often rely on national averages to model global trends, this research further extends the current understanding by offering finer spatial resolution and incorporating biological, behavioral, socioeconomic, and environmental variables within a unified data-driven framework.

### 4.1. Demographic and Temporal Disparities

The prominent racial disparities, with Black and Native American populations experiencing the highest CVD mortality, indicate the cumulative impact of socioeconomic disadvantages, environmental exposures, and healthcare access barriers [20,21]. The strong association between age and CVD mortality in the 65+ group reemphasizes the importance of early prevention strategies, such as lifestyle interventions and regular screenings, to mitigate risk accumulation over time [5]. Notably, the discrepancy of higher mortality in men across all age groups challenges the previous conclusion and necessitates the re-examination of gender-specific risk factors and interventions in CVD management [30,31]. The decline in CVD mortality from 2006 to 2019, followed by a significant rebound in 2020, is a critical notion, reflecting the COVID-19 impact on healthcare systems, lifestyle behaviors, and cardiovascular complications [1]. This warrants further investigation into its long-term effects and underscores the need for resilient healthcare systems capable of maintaining CVD management during public health crises.

### 4.2. Biological and Behavioral Determinants

Among individual-level exposures, COPD emerged as the dominant factor in CVD mortality, reaffirming the established bidirectional relationship involving shared inflammatory and vascular pathways [16]. Hypertension and stroke were also significant, advocating for patient-centered approaches to CVD and related comorbidities, incorporating multimodal interventions that target shared pathways to yield dual benefits [12,32,33]. Behavioral factors, including smoking, insufficient sleep, and medication non-adherence, ranked among the most influential contributors across all analyses, reinforcing their importance in CVD prevention [4,5,34]. Consistent with prior evidence, physical activity and cardiac rehabilitation participation were protective, highlighting the potential for lifestyle-based interventions to reduce risk [35,36]. These findings reinforce the need for cost-effective, practical solutions to enhance adherence to lifestyle interventions, particularly through targeted patient education [4,33,37].

Certain factors, such as alcohol consumption and obesity, exhibited counterintuitive correlation with CVD mortality, despite their traditional role as risk contributors. Their associations with CVD mortality should be interpreted cautiously, as they likely reflect residual confounding rather than true causal effects. For instance, regions with moderate alcohol consumption might concurrently benefit from higher income and healthcare access, providing alternative explanations to a direct cardioprotective effect [24]. Similarly, the association between obesity and CVD mortality aligns with the concept of an “obesity paradox”, wherein increased cardiac imaging and follow-up may allow for earlier detection, better medical follow-up, and improved survival in obese patients with underlying CVD [38]. Such observations underscore the need for longitudinal analyses to disentangle behavioral, socioeconomic, and clinical interactions.

### 4.3. Socioeconomic and Environmental Influences

Beyond individual behaviors, the study highlights the profound impact of socioeconomic and environmental inequalities on cardiovascular health. Counties with high social vulnerability and low family incomes correlated with elevated CVD mortality, confirming the central role of social determinants as fundamental contributors to health disparities [10,11,12]. Conversely, protective factors such as higher household income, park access, and community resilience point to the potential of structural improvements in reducing cardiovascular risks [35].

Environmental exposure to PM2.5 was identified as an important player in spatial disparities, supporting previous research linking long-term air pollution to elevated cardiovascular morbidity [7,18]. The prominence of PM2.5 and smoking status as strong factors highlights the interconnectedness of environmental and behavioral factors in cardiovascular health, emphasizing the critical need for integrated public health initiatives. Practically, these initiatives should simultaneously target respiratory and cardiovascular health through efforts like smoking cessation programs and the promotion of green infrastructures in vulnerable areas and communities.

### 4.4. Complementary Analytical Framework

The complementary application of traditional regression and dual ML models enhances the reproducibility, interpretability, and predictive strength of the findings. Stepwise regression provided transparent parameter estimates and statistical significance for identifying independent associations, whereas ML models captured nonlinear interactions and variable hierarchies. Together, these approaches move the evidence base beyond purely retrospective analysis toward proactive, geographically tailored prevention.

From a translational standpoint, the agreement between regression and ML models provides actionable confidence for public health policy. Both highlight COPD, medication non-adherence, smoking, and PM2.5 exposure as convergent targets for intervention. In practice, these results could inform data-driven prioritization of resource allocation and prevention efforts. For instance, the spatial patterns derived from regression residuals could help state and county health agencies visualize “hotspots” and prioritize counties with high-risk profiles for enhanced surveillance and subsidized medication access.

### 4.5. Public Health Implications

From a public health and policy perspective, the identified risk factors provide actionable entry points for mitigating cardiovascular disparities at regional and national scales. First, the strong influence of COPD, smoking, and PM2.5 underscores the need for integrated respiratory and cardiovascular health initiatives, such as joint screening programs, tobacco taxation, expansion of smoking cessation, pulmonary rehabilitation, and air-quality improvement in high-burden counties. Second, enhancing medication adherence and chronic disease management through mobile health technologies, pharmacist-led monitoring, and subsidized antihypertensive programs could directly reduce mortality, particularly in low-income and rural communities. Third, the spatial overlap between CVD mortality and social vulnerability suggests a role for place-based policy interventions, including investment in affordable housing, green spaces, and public transportation infrastructure to reduce environmental stressors. Finally, local health departments could use the ML-based predictions and spatial distributions from this study to create data-driven risk maps that guide prevention funding and health equity initiatives. Collectively, these strategies illustrate that mitigating CVD disparities requires not only individual-level behavior modification but also structural reforms addressing environmental, economic, and healthcare inequities.

## 5. Limitations

Despite offering valuable insights, this study has certain limitations that should be acknowledged. First, variability in county-level data quality may affect precision and cannot explain intra-county variations in risk factor exposure and health outcomes. Second, ecological design with reliance on publicly available datasets precludes inference at the individual level, and unmeasured confounders such as genetic predispositions or local healthcare characteristics may remain. Third, the cross-sectional nature prevents causal inference, restricting interpretation to associations rather than temporal relationships. Future research could benefit from longitudinal studies by integrating more granular individual-level data to track changes in risk factor prevalence and their impact on CVD mortality rates over time.

## 6. Conclusions

Management of CVD burden requires a multifaceted approach encompassing public health initiatives, clinical care, and policy interventions. The study herein provides a robust framework to understand the major linkage pathways to CVD mortality disparities across the U.S., offering actionable insights for the development of data-driven interventions to promote population health outcomes. Key associated factors, including COPD, smoking, PM2.5, and medication non-adherence, provide opportunities for targeted interventions. The persistent geographic disparities, particularly elevated mortality in the southeastern U.S. coinciding with areas of high social vulnerability, highlight the profound influence of systemic inequalities on cardiovascular health outcomes. The protective effects of structural factors such as income and healthcare access further emphasize the need for policies to address systemic inequities to reduce CVD burden. The significant 2020 rebound in CVD mortality signals the potential long-term consequences of public health crises like COVID-19 on cardiovascular health. In practical terms, this research advocates for a multi-pronged approach to mitigate CVD mortality disparities. Further investigation into the cost-effectiveness of tailored interventions in high-risk counties would be valuable for effectively guiding resource allocation. This should particularly focus on strategies that incorporate lifestyle modifications with pharmacological treatments to address environmental and socioeconomic determinants. Effective strategies may prioritize integrated initiatives with foci on (1) addressing key environmental and behavioral risk factors; (2) implementing primary prevention for patients with chronic conditions through increased physical activity, smoking reduction, and enhanced medication adherence support; (3) improving early detection and treatment via regular screenings for chronic diseases like COPD and hypertension; (4) mitigating socioeconomic and educational inequalities; and (5) developing innovative interventions such as mobile health technologies, telehealth services, and community-based care programs to enhance healthcare access and support for high-risk communities. By practicing data-driven interventions at the local and regional levels, it may help reduce the CVD burden throughout the nation. Future research efforts should explore longitudinal changes and assess the effectiveness of targeted interventions in mitigating their temporal effects on CVD outcomes, enabling more reliable long-term forecasting and the management of cardiovascular health.

## Figures and Tables

**Table 1:** Correlation of ranked risk factors to CVD mortality across U.S. counties.

Factor	R	*p*	N	Factor	R	*p*	N
COPD Prevalence	0.700	<0.0001	3054	Socioeconomic Vulnerability	0.534	<0.0001	3121
Current Smoker Status	0.650	<0.0001	3070	Population with Disability	0.526	<0.0001	3122
High Blood Pressure	0.644	<0.0001	3070	Adults No College Degree	0.513	<0.0001	3200
Less Sleeping < 7 h	0.644	<0.0001	3121	Alcohol Use	−0.509	<0.0001	3054
Population Living in Poverty	0.591	<0.0001	3128	Social Resilience	−0.533	<0.0001	3123
Food Stamp Percentage	0.547	<0.0001	3136	Median Household Income	−0.590	<0.0001	3128
Stroke Prevalence	0.540	<0.0001	3070				
Overall Vulnerability Rank	0.481	<0.0001	3121	Renin Angiotensin Antagonist NA	0.393	<0.0001	3147
Social Vulnerability	0.478	<0.0001	3113	High Cholesterol Prevalence	0.388	<0.0001	3070
Single-parent Households	0.478	<0.0001	3122	Air Quality PM2.5	0.383	<0.0001	3118
Asthma Prevalence	0.470	<0.0001	3054	Leisure-time Physical Inactivity	0.379	<0.0001	3070
Coronary Heart Disease	0.439	<0.0001	3070	Incremental Post-Acute Care Cost	0.374	<0.0001	3199
Diuretic Non-Adherence	0.428	<0.0001	3108	Population without HS Diploma	0.345	<0.0001	3200
Diagnosed Diabetes	0.417	<0.0001	3070	Family without Internet	0.339	<0.0001	3205
Mobile Housing Units	0.414	<0.0001	3122	Blood Pressure Medication Use	0.307	<0.0001	3070
Post-Acute Care Cost	0.411	<0.0001	3199	BRIC Resilience	−0.331	<0.0001	3123
Cardiac Rehabilitation Eligibility	0.401	<0.0001	3044	Park Access Percent	−0.336	<0.0001	3137
Blood Pressure Medication NA	0.397	<0.0001	3161	Housing-Infrastructural Resilience	−0.413	<0.0001	3123
DEHP in Water	0.396	<0.0001	123	Cardiac Rehabilitation Participation	−0.413	<0.0001	2399
Household Composition Disability	0.395	<0.0001	3121	Median Home Value	−0.453	<0.0001	3197

**Table 2:** Stepwise multiple regression of CVD mortality and highly correlated factors.

Coefficient Term	Coef	SE Coef	T-Value	*p*-Value	VIF
Constant	−0.018	0.234	−0.08	0.940	
Single-parent Households	0.593	0.219	2.71	0.007	3.03
Disability	0.434	0.210	2.07	0.039	2.73
Mobile-home Housing	−0.512	0.098	−5.25	0.000	2.29
Alcohol Use	−0.826	0.359	−2.30	0.021	2.53
Blood Pressure Medication Use	1.230	0.256	4.79	0.000	1.93
Cardiac Rehabilitation Eligibility	1.070	0.129	8.31	0.000	1.31
Diabetes	1.010	0.496	2.03	0.042	1.65
Food Stamp	0.364	0.164	2.22	0.026	3.59
Median Household Income	−0.458	0.071	−6.47	0.000	3.77
No College Degree	0.301	0.115	2.61	0.009	3.78
No High School Diploma	−1.060	0.177	−5.97	0.000	2.70
Air Quality PM2.5	4.720	0.423	11.15	0.000	1.41
Park Access	−0.072	0.029	−2.50	0.012	1.65
Less Sleeping < 7 h	0.931	0.287	3.25	0.001	3.09
Post-Acute Care Cost	0.019	0.005	3.66	0.000	1.43
Blood Pressure Medication Non-adherence	0.089	0.006	13.79	0.000	3.28
Smoking Status	4.480	0.308	14.55	0.000	3.92

## Data Availability

The data presented in this study were derived from publicly available resources provided by the CDC, accessed on 21 November 2024. These sources include: CDC Heart Disease & Stroke Interactive Atlas (http://nccd.cdc.gov/DHDSPAtlas/Reports.aspx); CDC National Environmental Public Health Tracking Network (https://ephtracking.cdc.gov/download); and the CDC National Center for Health Statistics (NCHS) (https://data.cdc.gov/browse). No new raw data were created. The processed, merged county-level dataset and associated analysis scripts supporting this article are available from the corresponding author upon reasonable request.

## Supplementary Materials

The following supporting information can be downloaded at: https://www.mdpi.com/article/10.3390/healthcare13222937/s1, Table S1: Correlation of various factors to CVD mortality rates across U.S. counties.
