# Supplementary material for: CVD Mortality Disparities with Risk Factor Associations Across U.S. Counties
Source: Healthcare (Basel). 2025 Nov 17;13(22):2937. doi: 10.3390/healthcare13222937 (PMC12652022; doi:10.3390/healthcare13222937)
Supplement: Supplementary file 1 [file healthcare-13-02937-s001.zip › healthcare-3948443-supplementary.pdf]

Supplementary Materials

Table S1: Correlation of various factors to CVD mortality rates across U.S. counties

Factor	R	*P*	N	Factor	R	*P*	N
COPD Prevalence	0.700	< 0.0001	3054	Vehicle Crashes Involving People	0.081	< 0.0001	3121
Current Smoker Status	0.650	< 0.0001	3070	Atrazine in Water	0.075	0.276	214
High Blood Pressure	0.644	< 0.0001	3070	Population per Neurologist	0.073	0.018	1059
Less Sleeping < 7 Hour	0.644	< 0.0001	3121	Population per CVD Physician	0.034	0.232	1212
Population Living in Poverty	0.591	< 0.0001	3128	Percent Cholesterol Screening	0.028	0.126	3070
Food Stamp Percentage	0.547	< 0.0001	3136	Housing of More People than Rooms	0.025	0.168	3122
Stroke Prevalence	0.540	< 0.0001	3070	TCE in Water	0.014	0.840	208
Socioeconomic Vulnerability	0.534	< 0.0001	3121	Air Domain Index	0.011	0.554	2769
Population with Disability	0.526	< 0.0001	3122	Water Domain Index	0.009	0.653	2769
Adults No College Degree	0.513	< 0.0001	3200	Sociodemographic Vulnerability	0.007	0.695	2769
Overall Vulnerability Rank	0.481	< 0.0001	3121	Population 65+	0.004	0.825	2769
Social Vulnerability	0.478	< 0.0001	3113	Environmental Quality Index	0.004	0.818	2769
Single-parent Households	0.478	< 0.0001	3122	Group Quarters	0.001	0.965	2769
Asthma Prevalence	0.470	< 0.0001	3054	More Units Housing	-0.000	0.993	2769
Coronary Heart Disease	0.439	< 0.0001	3070	Half Income on Housing	-0.001	0.975	3134
Diuretic Non-adherence	0.428	< 0.0001	3108	Land Domain Index	-0.001	0.977	2769
Diagnosed Diabetes	0.417	< 0.0001	3070	Community Capital Resilience	-0.003	0.881	3123
Mobile Housing Units	0.414	< 0.0001	3122	Built Domain Index	-0.005	0.797	2769
Post-Acute Care Cost	0.411	< 0.0001	3199	Physical Annual	-0.009	0.625	2769
Cardiac Rehabilitation Eligibility	0.401	< 0.0001	3044	Population 19-	-0.016	0.395	2769
Blood Pressure Medication NA	0.397	< 0.0001	3161	House Built 1980B	-0.013	0.452	3122
DEHP in Water	0.396	< 0.0001	123	Environmental Resilience Score	-0.029	0.103	3123
Household Composition Disability	0.395	< 0.0001	3121	Less English Speaker	-0.030	0.119	2769
Renin Angiotensin Antagonist NA	0.393	< 0.0001	3147	Land for Development - Open Space	-0.037	0.039	3095
High Cholesterol Prevalence	0.388	< 0.0001	3070	Land Covered by Water	-0.048	0.008	3095
Air Quality PM2.5	0.383	< 0.0001	3118	Uranium in Water	-0.056	0.335	301
Leisure-time Physical Inactivity	0.379	< 0.0001	3070	Land for Development - L Intensity	-0.065	0.000	3095
Incremental Post-Acute Care Cost	0.374	< 0.0001	3199	Public Transportation to Work	-0.066	0.000	3113
Population without HS Diploma	0.345	< 0.0001	3200	Radium in Water	-0.069	0.067	700
Family without Internet	0.339	< 0.0001	3205	Land for Development - H Intensity	-0.079	< 0.0001	3095
Blood Pressure Medication Use	0.307	< 0.0001	3070	Cancer Prevalence	-0.082	< 0.0001	3054
Cardiac Rehabilitation Completion	0.283	< 0.0001	1322	Land used for Agriculture - Crop	-0.085	< 0.0001	3095
Total Care Cost	0.282	< 0.0001	3199	Population per Neurosurgeon	-0.089	0.021	669
Cardiac Rehabilitation Completion	0.283	< 0.0001	1322	Hospitals with Cardiac Intensive Care	-0.090	< 0.0001	3209
Cardiac Rehabilitation Sessions	0.277	< 0.0001	3100	PCE in Water	-0.091	0.177	221
Sunlight and UV	0.277	< 0.0001	3200	Developed Imperviousness	-0.097	< 0.0001	3095
Family without Computer	0.268	< 0.0001	3122	Number of Hospitals	-0.104	< 0.0001	3209
House Built 1950A1980B	0.260	< 0.0001	3199	Walking to Work	-0.105	< 0.0001	3113
Incremental Total Care Cost	0.258	< 0.0001	3121	Economic Resilience Score	-0.108	< 0.0001	3123
Housing-Transportation Rank	0.251	< 0.0001	3136	Land for Development - M Intensity	-0.113	< 0.0001	3095
Cholesterol Medication NA	0.230	< 0.0001	3120	Hospitals with ER	-0.115	< 0.0001	3209
Population without Health Insurance	0.228	< 0.0001	2988	Health Device Reliant Populations	-0.120	< 0.0001	3122
Drug Poisoning Death Rate	0.225	< 0.0001	2974	Hospitals with Neurological Services	-0.123	< 0.0001	3209
Population per Primary Care Physician	0.225	< 0.0001	3122	Nitrates in Water	-0.128	< 0.0001	1440

Household without Vehicle	0.219	< 0.0001	3137	Driving to Work	-0.137	< 0.0001	3113
Urban-Rural Status	0.187	< 0.0001	3210	Incremental Outpatient Care Cost	-0.140	< 0.0001	3199
Number of Pharmacies & Drug Store	0.186	< 0.0001	1446	Hospitals with Cardiac Rehabilitation	-0.144	< 0.0001	3209
Disinfection H in Water	0.175	< 0.0001	3199	Outpatient Care Cost	-0.156	< 0.0001	3199
Inpatient Care Cost	0.174	< 0.0001	3095	Arsenic in Water	-0.168	< 0.0001	770
Land used for Agriculture - Pasture	0.166	< 0.0001	3112	Working from Home	-0.180	< 0.0001	3113
Time Driving to Work	0.166	< 0.0001	3200	House Built 1950B	-0.188	< 0.0001	3122
Income Inequality Gini Index	0.156	< 0.0001	1519	Households with Smartphone	-0.298	< 0.0001	3113
Disinfection T in Water	0.146	< 0.0001	3199	Radon Levels	-0.298	< 0.0001	805
Incremental Inpatient Care Cost	0.141	< 0.0001	3123	BRIC Resilience	-0.331	< 0.0001	3123
Institutional Resilience Score	0.132	< 0.0001	3123	Park Access Percent	-0.336	< 0.0001	3137
Minority Status Rank	0.132	< 0.0001	3121	Housing-Infrastructural Resilience	-0.413	< 0.0001	3123
Unemployment Rate	0.120	< 0.0001	3207	Cardiac Rehabilitation Participation	-0.413	< 0.0001	2399
Vehicle Crashes Involving No People	0.102	< 0.0001	3122	Median Home Value	-0.453	<0.0001	3197
Percent of Land Covered by Forest	0.102	< 0.0001	3095	Alcohol Use	-0.509	< 0.0001	3054
Obesity	0.090	< 0.0001	3070	Social Resilience	-0.533	< 0.0001	3123
Renter-occupied Housing Units	0.088	< 0.0001	3122	Median Household Income	-0.590	< 0.0001	3128
